# Human Immunodeficiency Virus 1 and Type I Interferons—Where Sex Makes a Difference

**DOI:** 10.3389/fimmu.2017.01224

**Published:** 2017-09-28

**Authors:** Susanne Maria Ziegler, Marcus Altfeld

**Affiliations:** ^1^Heinrich Pette Institute, Leibniz Institute for Experimental Virology, Hamburg, Germany; ^2^Institute of Immunology, University Medical Center Hamburg-Eppendorf, Hamburg, Germany

**Keywords:** type I interferon, human immunodeficiency virus 1, sex hormones, immune activation, sex differences, toll-like receptor 7

## Abstract

The human immunodeficiency virus (HIV)-1 epidemic continues to represent a global health problem that is over-proportionally affecting women from sub-Saharan Africa. Besides social and environmental factors, the modulation of immunological pathways by sex hormones and gene dosage effects of X chromosomal-encoded genes have been suggested to lead to differential outcomes in HIV-1 disease. Women present with lower HIV-1 loads early in infection. However, the progression to AIDS for the same level of viremia is faster in women than in men. Type I interferons (IFNs) play a prominent role in the control of HIV-1 transmission and replication. Continuous stimulation of type I IFNs in chronic viral infections can lead to increased levels of immune activation, which can be higher in HIV-1-infected women than in men. A role of steroid hormone signaling in regulating viral replication has been postulated, which might further account for sex differences observed in HIV-1 infections. Here, we review recent findings and current knowledge on sex-specific differences in HIV-1 infections.

## Introduction

Infections with human immunodeficiency virus (HIV)-1 still represent a global health problem with especially women and girls in sub-Saharan Africa being severely affected (1). The prevalence of HIV-1-infected girls and young women is twice as high as in men of same ages (2). While the overall survival does not differ between infected males and females (3, 4), women are at increased risk to acquire HIV-1 *via* heterosexual contact (5). It is now well established that sex-related biological factors affect HIV-1 disease manifestations and progression. During acute infection, HIV-1-infected females have lower HIV-1 viral load levels in comparison to men (6–9). Plasma viral load levels predict the size of the HIV-1 reservoir (10, 11), hence lower viral load levels in HIV-1-infected females might contribute to a smaller size of the HIV-1 DNA reservoir (12, 13). Type I interferons (IFNs) play a critical role in restricting viral replication through induction of host restriction factors and might contribute to this initial control of HIV-1 viremia. On the other hand, type I IFNs have been shown to increase systemic immune activation upon chronic viral infection (14, 15).

Several factors have been suggested to influence the sex bias observed in the manifestations of HIV-1 infections, including sex and gender differences in comorbidities as well as socioeconomic factors (16, 17). Dissecting biological mechanisms from socioeconomic factors represents a frequently challenging process. The elucidation of the biological mechanisms that underlie immunological differences between the sexes has become an emerging area of interest aimed at understanding the involvement of steroid hormones (18–20), the direct effects of X and Y chromosomal-linked factors (21–23) or epigenetic modifications that influence the composition and phenotype of immune cells, and the role of the microbiome (24). Recent reviews have already covered several aspects of sex-based differences in HIV-1 infection (16, 25–27). This review will focus on the latest findings contributing to our understanding of the impact of sex-based differences in type I IFNs on HIV-1 disease pathogenesis.

## Type I IFNs Control HIV-1 Replication

The process of HIV-1 transmission through the mucosa of the female genital tract is comparatively unproductive (28). The innate immune system plays a major role in sensing incoming HIV-1 to initiate antiviral responses (29). HIV-1 can be recognized by multiple nucleic acid sensors, including toll-like receptors (TLRs) 3, 7, 8, and 9 (30), IFN-inducible protein (IFI) 16 (31), and cyclic GMP-AMP synthase (32). Recognition of HIV-1 by pattern recognition receptors leads to induction of antiviral and proinflammatory responses *via* JAK/STAT signaling, NF-κB, and interferon regulatory factors (IRFs) (33), ultimately resulting in the production of cytokines, chemokines, and type I IFNs. Type I IFNs are the first line of defense against viral infections, as they are capable of mediating immunregulatory, growth-inhibitory, and antiviral activities (34), through the induction of a multitude of interferon-stimulated genes (ISGs) that encode for antiviral proteins. In humans, the type I IFN family of genes consists of 13 highly homologous IFNα subtypes, IFNβ, IFNε, IFNκ, and IFNω (35). All type I IFNs bind to the same IFNα/β receptor (IFNAR) composed of two subunits IFNAR1 and IFNAR2, with varying affinities for the two subunits (36, 37). IFNARs are expressed on most cell types and their activation induces the transcription of ISGs. Type I IFNs play an expanding role in restricting HIV-1 replication (38). Productive infections in a new host were shown to be established by HIV-1 strains (transmitted founder viruses) that were much less susceptible to the antiviral effects of type I IFNs *in vitro* (39–41). In line with these antiviral effects of type I IFNs that have to be overcome by transmitted founder viruses, one recent study demonstrated that vaginal IFNβ administration in rhesus macaques upregulated the expression of ISGs in vaginal suspensions and prevented SHIV-1 acquisition (42). The distinct capacities of the 13 different IFNα subtypes in controlling retroviral infections have been studied using humanized mouse models, suggesting that IFNα8 and IFNα14 suppress HIV-1 replication significantly better than other IFNα subtypes (43). Garcia-Minambres et al. recently described another, less prominent, type I IFN important in HIV-1 restriction (44). IFNε is expressed in the female genital tract of mice and humans and induces several HIV-1 restriction factors in T cells, with levels and antiviral effects similar to IFNα-mediated induction. IFNε reduced HIV-1 replication *in vitro* and HIV-1 strains that were grown in the presence of IFNε were less infectious than HIV-1 that was produced in the absence of IFNε (44). Interestingly, IFNε expression in the female reproductive tract is hormone dependent, with the highest expression during the proliferative phase of the menstrual cycle, when estrogen levels are peaking (45). During the secretory phase, which is characterized by a peak in progesterone, IFNε levels decline (45). This decrease in IFNε in the presence of high progesterone might play a role in the suggested increase in HIV-1 acquisition in females that take long-acting contraceptives (46–50). Taken together, type I IFN levels that are increased within the first weeks of HIV-1 infection induce expression of HIV-1 restriction factors, which play an important role in restricting HIV-1 spread and replication early in infection.

## Sex Differences in Type I IFN Production upon TLR7-Mediated Recognition of ssRNA

Plasmacytoid dendritic cells (pDCs) are innate immune cells that are specialized to produce type I IFNs and mediate cross-talk between innate and adaptive immunity. Multiple studies have contributed important knowledge on the role of pDCs in mediating sex-specific, HIV-1-associated immune activation. pDCs from females produce higher levels of IFNα upon TLR7 stimulation using HIV-1 derived ssRNA (20) or synthetic ligands (18, 19, 51, 52) in comparison to men. In addition, we recently showed that pDCs from females express significantly higher mRNA levels of all 13 IFNα subtypes and IFNβ after TLR7-stimulation of peripheral blood mononuclear cells (PBMCs) (52). The mRNA expression levels of IFNα in pDCs were correlated with the expression levels of IFNβ in pDCs after TLR7 stimulation, but were independent from secondary signaling *via* IFNAR (52), as reported for TLR9 signaling or type I IFN production by other cell types than pDCs (53–55). Although IFNα produced by pDCs is able to inhibit HIV-1 replication by exerting growth-inhibitory and antiviral activities, the continuous exposure of pDCs to HIV-1 has opposing effects. HIV-1 recognition by pDCs leads to the expression of low levels of maturation molecules on pDCs and therefore skews pDCs toward a partially matured, persistently IFNα-secreting phenotype stimulating only weak T cell responses (56). The effect of chronic exposure to IFNα was recently studied in mice, showing that chronic IFNα stimulation was sufficient to suppress specific CD8 T cell responses to vaccinia virus infection by inducing the accumulation of suppressive Ly6Chi monocytes (57). HIV-1 in turn has been suggested to impair TLR7-mediated IFNα production by pDCs, potentially by binding to CD303 receptors expressed on pDCs (58).

TLR7 is encoded on the X chromosome (22). X chromosome inactivation (XCI) of one of the two female X chromosomes ensures dosage compensation in female mammals, in contrast to male mammals harboring only one X chromosome. Most genes on the X chromosome are assumed to be transcriptionally silent; however, the process of XCI is dynamic and relies on dosage-dependent activators. A number of genes are known to escape XCI and are expressed from both the active and inactive X chromosome (59–62). So far, there is no evidence for a higher expression of TLR7 genes in pDCs from women in humans (51, 63), though TLR7 gene duplication has been associated with autoreactive B cell responses in mice (64) and increased levels of TLR7 and TLR8 have been found in PBMCs of patients with the primary Sjögren’s syndrome, an autoimmune disease with a female sex imbalance (65). Taken together, further research investigating the molecular mechanisms underlying TLR7 expression and regulation in pDCs will help to dissect the role of TLR7 signaling and its contribution to the sex bias in type I IFN production in HIV-1-infected individuals.

## Impact of Hormones on HIV-1 Disease

Steroid hormones have also been shown to modulate TLR-dependent responses of pDCs in humans. Estrogens and estrogen receptor-dependent regulations play key roles in dendritic cell development and function (66). pDCs from postmenopausal women display reduced TLR7-responsiveness and IFNα production in comparison to women of reproductive age. Estrogen replacement therapy of postmenopausal women increased the percentage of IFNα-producing pDCs after TLR7 and TLR9 stimulation (18). *In vitro* blockage of ER signaling during pDC-differentiation dampened the IFNα response of pDCs after TLR7 stimulation (63). IFNα-induction by pDCs is regulated by IRFs at the transcriptional level (67). IRF5 is one of the central mediators of TLR7 signaling (68, 69) and has recently been shown to be expressed at higher basal levels in pDCs from females in comparison to males (19). While upregulation of IRF5 levels in pDCs resulted in increased IFNα secretion by pDCs, genetic ablation of the *Esr1* gene reduced IRF5 mRNA expression in pDCs and subsequent IFNα production in response to TLR7 stimulation (19).

Sex hormones have also been reported to directly modulate host factors that play a role in HIV-1 acquisition (25). Generally, progesterone increases susceptibility to viral infections whereas estrogen protects against HIV-1 acquisition (70, 71). Studies of rhesus macaques support this general concept, as simian immunodeficiency virus (SIV) susceptibility was highest in the luteal phase of the menstrual cycle (high progesterone) compared with the follicular phase (high estrogen) (72). Several studies report an increase in expression of HIV-1 receptors on cervical CD4 T cells mediated by progesterone (73–75), supporting the reported increase in HIV-1 acquisition. Nevertheless, one *in vitro* study suggests that progesterone decreases the upregulation of CCR5 on CD4 T cells in peripheral blood (76). Furthermore, Meditz et al. showed that postmenopausal women, who have lower levels of progesterone, have elevated cervical CCR5 expression, which may increase their risk for HIV-1 acquisition (77). Estrogen exerts several biphasic effects on cells of the immune system, including modulation of T helper 1 versus T helper 2 cell differentiation (22) and expansion of regulatory T cells (78). Animal studies have shown that topical estrogen protects against vaginal SIV transmission in rhesus macaques (79). Estrogen was shown to downregulate the susceptibility of CD4 T cells and macrophages to HIV-1 *in vitro* (80), yet some studies suggest that *in vivo* hormone treatment with estrogens and antiandrogens in men to female transsexuals upregulates CCR5 on CD4 T cells over time (81), and also in mice (82).

The direct influence of steroid hormones on viral replication capacity of HIV-1 has been investigated in a few studies. Estradiol and progesterone can potentially regulate HIV-1 replication by directly altering HIV-1 transcriptional activation (83); however, the current results are controversial. In one study, *in vitro* estradiol treatment was shown to inhibit production of HIV-1 (84), while another study suggested an estrogen-based increase of transcriptional activity of HIV-1 LTRs (85). In addition, hormonal effects on HIV-1 replication were shown to be donor and HIV-1 subtype specific (86). Further studies are required to determine how the hormonal milieu might shape the outcome of HIV-1 infection *in vivo* and to identify the underlying molecular mechanisms.

## Sex Differences in Immune Activation in Chronic Retroviral Infections of Mammals

It is now well established that systemic immune activation is a strong predictor for HIV-1 disease progression to AIDS. Consistent with the finding that innate immune function is linked to higher responses to TLR7 stimulation of pDCs and subsequent type I IFN production, HIV-1-infected women have higher expression levels of ISGs than men for the same level of viral load (87). Furthermore, the surface expression levels of the common IFN-α/β receptor subunit 2 (IFNAR2) are significantly higher on pDCs from females in comparison to males and tends to be higher on other antigen-presenting cells from females as well (52). The increase in ISG expression in female HIV-1-infected individuals might explain increased levels of T cell activation (CD4/8^+^ CD38^+^ HLA-DR^+^) in HIV-1-infected females in comparison to male HIV-1-infected individuals for the same level of viral load (20). Higher expression levels of IFNAR2 might contribute to the increased levels of ISG expression in females in comparison to males. In addition, higher levels of IFNα have been associated with decreased CD4 counts (88), which is in line with one study showing that increased expression of ISGs in CD4 T cells was associated with CD4 T cell depletion in HIV-1-infected individuals (89). The importance of type I IFNs in HIV-1 infections, as well as the timing of their effects, was studied in SIV infections of rhesus macaques (90). Blockage of IFN α/β receptors in rhesus macaques reduced antiviral gene expression and reservoir size during acute infections; however, continued IFNα2a treatment led to a desensitisation toward type I IFNs, resulting in decreased antiviral gene expression, increased reservoir size and accelerated CD4 T cell loss (90). Several mechanisms that underlie the association of immune activation and progression to AIDS have been proposed (14). Overall, it appears that the timing of type I IFN-induced innate response to HIV/SIV infection largely influences overall disease pathogenesis.

Several markers of inflammation and immune activation, such as C-reactive protein, sCD14, IL-6, and TNFα, have been associated with increased progression and mortality in HIV-1 infections (91–93). A novel non-human primate model for the investigation of sex differences in HIV-1 disease progression was recently used to study sex differences in local innate immune activation and gut microbiota (94). Viral load, disease progression, microbiota, and immunological parameters were studied in rhesus macaques infected with SHIV-1. This non-human primate model showed the same sex bias in SHIV-1 infection as observed for HIV-1 in humans and linked an earlier and more robust proinflammatory immune response as well as increased expansion of Proteobacteria in the female rectal mucosa to the increased disease susceptibility in female macaques (94).

A recent longitudinal study investigated sex differences in inflammatory and immune activation markers in HIV-1-infected women and men after initiation of combination antiretroviral treatment (cART). After cART initiation, women experienced less cART-associated reduction in inflammation and immune activation, indicated by higher levels of IFNγ and TNFα 48 weeks after initiation of therapy (95). Another study analyzed sex differences in soluble markers in plasma and cerebrospinal fluid (CSF) in HIV-1-infected treatment-naive individuals with or without cognitive impairment from Thailand. Soluble markers were quantified before and 48 weeks after cART initiation to identify variations in markers that may contribute to differences in disease progression (96). In chronic untreated HIV-1 infection, up to 50% of infected individuals have been reported to have cognitive impairment, commonly termed HIV-1-associated neurological disorders (97). Treatment naive, HIV-1-infected women with impaired cognition had elevated levels of neopterin and TNF-RII, both correlative markers for HIV-1 progression and efficiency of cART, compared to women with normal cognition in both plasma and CSF, whereas no associations were observed between these markers and cognition in men. Furthermore, sex-specific differences in the levels of a number of markers, including MCP-1, IL-8, IL-10, I-FABP, and sCD14, were detected in response to treatment (96). This study therefore proposed sex-specific differences in markers that were associated with cognitive impairment and chronic inflammation. Overall, these data suggest that changes in soluble markers vary before and after initiation of antiretroviral therapy between chronically HIV-1-infected women and men and might be predictive for HIV-1 disease outcomes.

## Conclusion

The precise role of type I IFNs in HIV-1 disease progression still remains insufficiently understood. The outcomes of type I IFNs on disease outcome are multifactorial and appear to depend on timing and extend of the induction (local versus systemic), as well as on the specific IFNα subtypes that are induced. Sex differences in type I IFN responses are now widely accepted; however, the underlying mechanisms that lead to sex-based differences in type I IFN production and their impact on infectious diseases such as HIV-1, but also on autoimmune diseases, remain less well understood. The frequent under-representation of women in clinical studies furthermore impedes research progress in that area. Thoughtful designed experimental and clinical studies will have to fill in the current gaps in knowledge concerning the impact of chromosomal effects and/or hormonal influences on innate immune responses and the subsequent consequences for HIV-1 disease progression.

## Author Contributions

All authors have made a substantial, direct, and intellectual contribution to the work and approved it for publication.

## Conflict of Interest Statement

The authors declare that the research was conducted in the absence of any commercial or financial relationships that could be construed as a potential conflict of interest.
